# 1,2-Bis(2-bromo­benz­yl)diselane

**DOI:** 10.1107/S1600536810036676

**Published:** 2010-09-18

**Authors:** Guoxiong Hua, Amy L. Fuller, Alexandra M. Z. Slawin, J. Derek Woollins

**Affiliations:** a Department of Chemistry, University of St Andrews, St Andrews KY16 9ST, Scotland

## Abstract

In the title compound, C_14_H_12_Br_2_Se_2_, the Se—Se bond length [2.3034 (9) Å] is similar to those in diphenyl diselenide [2.3066 (7) and 2.3073 (10) Å] and shorter than that in 1,8-diselenona­phthalene [2.0879 (8)Å]. The mol­ecule adopts a classical *gauche* conformation.

## Related literature

Related structures are: diphenyl diselenide (Fuller *et al.*, 2010 ▶); di(2-bromo­meth­yl)phenyl­diselenide (Lari *et al.*, 2009 ▶) and 1,8 diseleno-naphthalene (Aucott *et al.*, 2004 ▶).
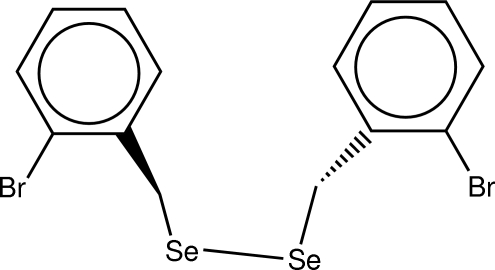


## Experimental

### 

#### Crystal data

C_14_H_12_Br_2_Se_2_
*M* *_r_* = 497.98
Monoclinic, 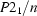

*a* = 10.873 (3) Å
*b* = 9.002 (2) Å
*c* = 15.714 (4) Å
β = 106.102 (6)°
*V* = 1477.9 (6) Å^3^
*Z* = 4
Mo *K*α radiation
μ = 10.41 mm^−1^
*T* = 125 K
0.15 × 0.09 × 0.09 mm

#### Data collection

Rigaku Saturn70 CCD diffractometer
Absorption correction: multi-scan *CrystalClear* (Rigaku Americas and Rigaku, 2009 ▶) *T*_min_ = 0.153, *T*_max_ = 0.392
9089 measured reflections
3124 independent reflections
2671 reflections with *F* ^2^ > 2σ(*F* ^2^)
*R*_int_ = 0.058

#### Refinement

*R*[*F* ^2^ > 2σ(*F* ^2^)] = 0.042
*wR*(*F* ^2^) = 0.082
*S* = 1.22
2939 reflections
163 parameters
H-atom parameters constrained
Δρ_max_ = 0.76 e Å^−3^
Δρ_min_ = −0.61 e Å^−3^

### 

Data collection: *CrystalClear* (Rigaku Americas and Rigaku, 2009 ▶); cell refinement: *CrystalClear*; data reduction: *CrystalClear*; program(s) used to solve structure: *SHELXS97* (Sheldrick, 2008 ▶); program(s) used to refine structure: *SHELXL97* (Sheldrick, 2008 ▶); molecular graphics: *CrystalStructure* (Rigaku Americas and Rigaku, 2010 ▶); software used to prepare material for publication: *CrystalStructure*.

## Supplementary Material

Crystal structure: contains datablocks global, I. DOI: 10.1107/S1600536810036676/bt5357sup1.cif

Structure factors: contains datablocks I. DOI: 10.1107/S1600536810036676/bt5357Isup2.hkl

Additional supplementary materials:  crystallographic information; 3D view; checkCIF report

## Figures and Tables

**Table:** 

Se1—C1	1.987 (5)
Se1—Se2	2.3034 (8)
Se2—C8	1.986 (5)

**Table:** 

C1—Se1—Se2—C8	88.8 (2)

**Fig. 1.**
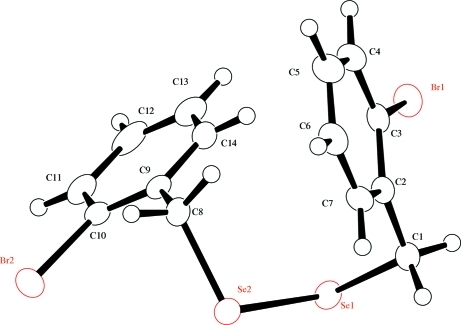
Anisotropic displacement ellipsoid plot of the title compound.

## supplementary crystallographic information

### Comment

We have recently reported (Fuller *et al.*, 2010) on the remarkable crystallization of PhSeSePh as one isomer. Here, the title compound (figure 1) is obtained as a mixture of both hands, the Se—Se bondlength, 2.3034 (9), is similar to that in diphenyldiselenide [2.3066 (7), 2.3073 (10) Å; Fuller *et al.*, 2010] and shorter than that in 1,8-diselenonaphthalene [2.0879 (8)Å; Aucott *et al.*, 2004).

### Experimental

To a solution of 2-bromobenzyl bromide (15.0 g, 60 mmol) in 150 ml of dry ethanol was added potassium selenocyanate (9.5 g, 65.0 mmol) at ambient temperature. The mixture was stirred for 2 h. Then an equeous solution of NaOH (4.8 g, 120 mmol in 200 ml of water) was added to the mixture and was continued stirring for another 2 h. After extracted by dichloromethane (300 ml) and washed three times with water (100 mLx3), the organic layer was dried over MgSO_4_ overnight. The organic residue was further purified by silica gel column (dichloromethane as eluent) to give a bright yellow solid (14.5 g) in 97% yield. Selected IR (KBr, cm^-1^): 2985(w), 1563(*m*), 1469(*m*), 1436(*m*), 1413(*m*), 1334(*m*), 1170(*m*), 1022(*s*), 755(*versus*), 718(*m*), 657(*m*), 589(*m*). ^1^H NMR (CD_2_Cl_2_, d), 7.54 (dd, *J*(H,H) = 8.0 Hz, 2H, ArH), 7.29–7.09 (m, 6H, ArH), 4.00 (s, 4H, CH_2_) p.p.m.. ^13^C NMR (CD_2_Cl_2_, d), 138.6, 133.1, 131.0, 128.9, 127.5, 124.3, 33.4 p.p.m.. ^77^Se NMR (CD_2_Cl_2_, d), 398.6 p.p.m.. MS (EI^+^, m/*z*), 498 [*M*]^+^. Accurate mass measurement [EI^+^, m/*z*]: 489.7664 [*M*]^+^, calculated mass for C_14_H_12_Br_2_^76^Se_2_: 489.7685. Anal. Calcd. for C_14_H_12_Br_2_Se_2_: C, 33.78; H, 2.43. Found: C, 33.92; H, 2.62.

### Refinement

All H atoms were included in calculated positions and refined as riding atoms with U\ĩso\~(H) = 1.5 U\~eq\~. The highest peak in the difference map is 1.12 Å from atom Se2.

### Crystal data

**Table:**

C_14_H_12_Br_2_Se_2_	*F*(000) = 936.00
*M**_r_* = 497.98	*D*_x_ = 2.238 Mg m^−^^3^
Monoclinic, *P*2_1_/*n*	Mo *K*α radiation, λ = 0.71075 Å
Hall symbol: -P 2yn	Cell parameters from 4976 reflections
*a* = 10.873 (3) Å	θ = 2.0–26.4°
*b* = 9.002 (2) Å	µ = 10.41 mm^−^^1^
*c* = 15.714 (4) Å	*T* = 125 K
β = 106.102 (6)°	Prism, colorless
*V* = 1477.9 (6) Å^3^	0.15 × 0.09 × 0.09 mm
*Z* = 4	

### Data collection

**Table:**

Rigaku Saturn70 CCD diffractometer	2671 reflections with *F*^2^ > 2σ(*F*^2^)
Detector resolution: 14.629 pixels mm^-1^	*R*_int_ = 0.058
ω scans	θ_max_ = 26.4°
Absorption correction: multi-scan *CrystalClear* (Rigaku Americas and Rigaku, 2009)	*h* = −9→13
*T*_min_ = 0.153, *T*_max_ = 0.392	*k* = −11→9
9089 measured reflections	*l* = −16→19
3124 independent reflections	

### Refinement

**Table:**

Refinement on *F*^2^	Secondary atom site location: difference Fourier map
*R*[*F*^2^ > 2σ(*F*^2^)] = 0.042	Hydrogen site location: inferred from neighbouring sites
*wR*(*F*^2^) = 0.082	H-atom parameters constrained
*S* = 1.22	*w* = 1/[σ^2^(*F*_o_^2^) + (0.0253*P*)^2^ + 2.0566*P*] where *P* = (*F*_o_^2^ + 2*F*_c_^2^)/3
2939 reflections	(Δ/σ)_max_ = 0.001
163 parameters	Δρ_max_ = 0.76 e Å^−^^3^
0 restraints	Δρ_min_ = −0.61 e Å^−^^3^
Primary atom site location: structure-invariant direct methods	

### Special details

**Table:**

Geometry. ENTER SPECIAL DETAILS OF THE MOLECULAR GEOMETRY
Refinement. Refinement was performed using all reflections. The weighted *R*-factor (*wR*) and goodness of fit (*S*) are based on *F*^2^. *R*-factor (gt) are based on *F*. The threshold expression of *F*^2^ > 2.0 σ(*F*^2^) is used only for calculating *R*-factor (gt).

### Fractional atomic coordinates and isotropic or equivalent isotropic displacement parameters (Å^2^)

**Table:**

	*x*	*y*	*z*	*U*_iso_*/*U*_eq_	
Br(1)	0.94202 (5)	0.82306 (5)	0.39444 (3)	0.02997 (15)	
Br(2)	0.24435 (5)	0.89337 (5)	0.36824 (4)	0.03177 (15)	
Se(1)	0.63257 (5)	0.62609 (5)	0.28455 (3)	0.02562 (14)	
Se(2)	0.48881 (5)	0.58891 (5)	0.36604 (3)	0.02668 (14)	
C(1)	0.7876 (5)	0.5176 (5)	0.3532 (3)	0.0245 (10)	
C(2)	0.8288 (5)	0.5639 (5)	0.4477 (3)	0.0213 (10)	
C(3)	0.8944 (5)	0.6951 (5)	0.4770 (3)	0.0222 (10)	
C(4)	0.9269 (5)	0.7397 (5)	0.5647 (3)	0.0275 (11)	
C(5)	0.8920 (5)	0.6504 (6)	0.6263 (3)	0.0313 (11)	
C(6)	0.8282 (5)	0.5183 (5)	0.5996 (3)	0.0279 (11)	
C(7)	0.7967 (5)	0.4760 (5)	0.5117 (3)	0.0241 (10)	
C(8)	0.5260 (5)	0.7583 (5)	0.4503 (3)	0.0247 (10)	
C(9)	0.5175 (5)	0.9065 (5)	0.4057 (3)	0.0223 (10)	
C(10)	0.4031 (5)	0.9784 (5)	0.3655 (3)	0.0217 (10)	
C(11)	0.3992 (6)	1.1154 (5)	0.3247 (3)	0.0289 (12)	
C(12)	0.5135 (6)	1.1812 (5)	0.3217 (3)	0.0359 (14)	
C(13)	0.6279 (6)	1.1126 (5)	0.3605 (4)	0.0364 (14)	
C(14)	0.6297 (5)	0.9771 (5)	0.4025 (3)	0.0298 (11)	
H(1a)	0.7695	0.4097	0.3499	0.029*	
H(1b)	0.8582	0.5359	0.3261	0.029*	
H(4)	0.9722	0.8299	0.5825	0.033*	
H(5)	0.9119	0.6802	0.6866	0.038*	
H(6)	0.8059	0.4562	0.6420	0.033*	
H(7)	0.7523	0.3852	0.4943	0.029*	
H(8a)	0.4648	0.7561	0.4866	0.030*	
H(8b)	0.6132	0.7460	0.4907	0.030*	
H(11)	0.3198	1.1635	0.2991	0.035*	
H(12)	0.5123	1.2740	0.2928	0.043*	
H(13)	0.7059	1.1581	0.3585	0.044*	
H(14)	0.7096	0.9312	0.4297	0.036*	

### Atomic displacement parameters (Å^2^)

**Table:**

	*U*^11^	*U*^22^	*U*^33^	*U*^12^	*U*^13^	*U*^23^
Br(1)	0.0302 (3)	0.0269 (3)	0.0381 (3)	−0.0038 (2)	0.0184 (3)	0.0042 (2)
Br(2)	0.0225 (3)	0.0318 (3)	0.0397 (3)	−0.0011 (2)	0.0065 (2)	−0.0027 (2)
Se(1)	0.0286 (3)	0.0285 (3)	0.0192 (2)	0.0043 (2)	0.0057 (2)	−0.00085 (18)
Se(2)	0.0256 (3)	0.0213 (2)	0.0342 (3)	−0.00192 (19)	0.0101 (2)	−0.00485 (19)
C(1)	0.022 (3)	0.022 (2)	0.030 (3)	0.003 (2)	0.007 (2)	0.0001 (19)
C(2)	0.019 (3)	0.020 (2)	0.026 (2)	0.0021 (19)	0.008 (2)	0.0020 (18)
C(3)	0.018 (3)	0.022 (2)	0.029 (3)	0.0021 (19)	0.010 (2)	0.0044 (19)
C(4)	0.023 (3)	0.030 (2)	0.029 (3)	−0.004 (2)	0.007 (2)	0.000 (2)
C(5)	0.028 (3)	0.041 (3)	0.021 (3)	0.000 (2)	0.000 (2)	0.001 (2)
C(6)	0.026 (3)	0.030 (2)	0.028 (3)	0.002 (2)	0.007 (2)	0.010 (2)
C(7)	0.020 (3)	0.020 (2)	0.031 (3)	−0.0019 (19)	0.004 (2)	0.0018 (19)
C(8)	0.023 (3)	0.028 (2)	0.022 (2)	0.006 (2)	0.005 (2)	−0.0009 (19)
C(9)	0.026 (3)	0.021 (2)	0.022 (2)	−0.0023 (19)	0.010 (2)	−0.0063 (18)
C(10)	0.025 (3)	0.022 (2)	0.020 (2)	−0.0031 (19)	0.008 (2)	−0.0052 (18)
C(11)	0.040 (3)	0.023 (2)	0.025 (3)	0.004 (2)	0.011 (2)	−0.0035 (19)
C(12)	0.065 (4)	0.020 (2)	0.032 (3)	−0.004 (3)	0.029 (3)	−0.005 (2)
C(13)	0.045 (4)	0.029 (3)	0.045 (3)	−0.015 (3)	0.028 (3)	−0.015 (2)
C(14)	0.025 (3)	0.032 (3)	0.035 (3)	−0.002 (2)	0.013 (2)	−0.011 (2)

### Geometric parameters (Å, °)

**Table:**

Br1—C3	1.911 (4)	C6—H6	0.9500
Br2—C10	1.899 (5)	C7—H7	0.9500
Se1—C1	1.987 (5)	C8—C9	1.498 (6)
Se1—Se2	2.3034 (8)	C8—H8A	0.9900
Se2—C8	1.986 (5)	C8—H8B	0.9900
C1—C2	1.487 (6)	C9—C14	1.389 (7)
C1—H1A	0.9900	C9—C10	1.389 (7)
C1—H1B	0.9900	C10—C11	1.385 (6)
C2—C3	1.391 (6)	C11—C12	1.389 (8)
C2—C7	1.398 (6)	C11—H11	0.9500
C3—C4	1.384 (7)	C12—C13	1.371 (8)
C4—C5	1.389 (7)	C12—H12	0.9500
C4—H4	0.9500	C13—C14	1.385 (7)
C5—C6	1.383 (7)	C13—H13	0.9500
C5—H5	0.9500	C14—H14	0.9500
C6—C7	1.382 (7)		
			
C1—Se1—Se2	103.36 (13)	C2—C7—H7	119.3
C8—Se2—Se1	102.41 (14)	C9—C8—Se2	113.4 (3)
C2—C1—Se1	112.3 (3)	C9—C8—H8A	108.9
C2—C1—H1A	109.1	Se2—C8—H8A	108.9
Se1—C1—H1A	109.1	C9—C8—H8B	108.9
C2—C1—H1B	109.1	Se2—C8—H8B	108.9
Se1—C1—H1B	109.1	H8A—C8—H8B	107.7
H1A—C1—H1B	107.9	C14—C9—C10	117.1 (4)
C3—C2—C7	116.7 (4)	C14—C9—C8	118.9 (5)
C3—C2—C1	123.6 (4)	C10—C9—C8	124.0 (4)
C7—C2—C1	119.6 (4)	C11—C10—C9	122.2 (5)
C4—C3—C2	122.8 (4)	C11—C10—Br2	117.3 (4)
C4—C3—Br1	117.3 (3)	C9—C10—Br2	120.5 (3)
C2—C3—Br1	119.8 (3)	C10—C11—C12	118.9 (5)
C3—C4—C5	118.8 (5)	C10—C11—H11	120.6
C3—C4—H4	120.6	C12—C11—H11	120.6
C5—C4—H4	120.6	C13—C12—C11	120.2 (5)
C6—C5—C4	119.9 (5)	C13—C12—H12	119.9
C6—C5—H5	120.1	C11—C12—H12	119.9
C4—C5—H5	120.1	C12—C13—C14	119.9 (5)
C7—C6—C5	120.3 (4)	C12—C13—H13	120.0
C7—C6—H6	119.8	C14—C13—H13	120.0
C5—C6—H6	119.8	C13—C14—C9	121.6 (5)
C6—C7—C2	121.4 (4)	C13—C14—H14	119.2
C6—C7—H7	119.3	C9—C14—H14	119.2
			
C1—Se1—Se2—C8	88.8 (2)	Se1—Se2—C8—C9	55.2 (4)
Se2—Se1—C1—C2	−53.2 (3)	Se2—C8—C9—C14	−101.0 (4)
Se1—C1—C2—C3	−77.5 (5)	Se2—C8—C9—C10	78.0 (5)
Se1—C1—C2—C7	99.9 (4)	C14—C9—C10—C11	−0.8 (6)
C7—C2—C3—C4	−0.5 (7)	C8—C9—C10—C11	−179.8 (4)
C1—C2—C3—C4	176.9 (4)	C14—C9—C10—Br2	−178.7 (3)
C7—C2—C3—Br1	−179.8 (3)	C8—C9—C10—Br2	2.2 (6)
C1—C2—C3—Br1	−2.4 (6)	C9—C10—C11—C12	1.7 (7)
C2—C3—C4—C5	−0.3 (7)	Br2—C10—C11—C12	179.8 (3)
Br1—C3—C4—C5	179.0 (4)	C10—C11—C12—C13	−1.4 (7)
C3—C4—C5—C6	1.2 (8)	C11—C12—C13—C14	0.2 (7)
C4—C5—C6—C7	−1.3 (8)	C12—C13—C14—C9	0.8 (7)
C5—C6—C7—C2	0.5 (8)	C10—C9—C14—C13	−0.5 (7)
C3—C2—C7—C6	0.4 (7)	C8—C9—C14—C13	178.6 (4)
C1—C2—C7—C6	−177.1 (4)		
